# Mutant, wild type, or overall p53 expression: freedom from clinical progression in tumours of astrocytic lineage

**DOI:** 10.1038/sj.bjc.6602161

**Published:** 2004-10-19

**Authors:** F S Pardo, D W Hsu, R Zeheb, J T Efird, P G Okunieff, D M Malkin

**Affiliations:** 1 Division of Radiation Oncology, Radiation and Cellular Biology Laboratory, Department of Radiology, University of California, San Diego, CA, USA; 2 Department of Radiation Oncology, Massachusetts General Hospital/Harvard Medical School, Boston, MA, USA; 3 Department Pathology, Massachusetts General Hospital/Harvard Medical School, Boston, MA, USA; 4 MGH Cancer Center, Massachusetts General Hospital/Harvard Medical School, Boston, MA, USA; 5 Oncogene Science, Inc., Uniondale, NY, USA; 6 Department of Epidemiology and Biostatistics, Stanford University Medical Center, Stanford, CA, USA; 7 Department of Radiation Oncology, University of Rochester, Rochester, NY, USA; 8 Division of Oncology, The Hospital for Sick Children, Toronto, Ontario, Canada

**Keywords:** brain tumour, glioma survival, radiation therapy

## Abstract

Abnormalities of the p53 tumor-suppressor gene are found in a significant proportion of astrocytic brain tumours. We studied tumour specimens from 74 patients evaluated over 20 years at the Massachusetts General Hospital, where clinical outcome could be determined and sufficient pathologic material was available for immunostaining. p53 expression studies employed an affinity-purified p53 monoclonal antibody, whose specificity was verified in absorption studies and, in a minority of cases, a second antibody recognising a different epitope of p53. Significant overexpression of p53 protein was found in 48% of the 74 tumours included in this series and high levels of expression were associated with higher mortality from astrocytic tumours (*P*<0.001, log rank). Multivariate analyses revealed that immunohistochemically detected p53 was an independent marker of shortened progression-free and overall actuarial survival in patients with astrocytic tumours, suggesting that increased expression of p53 plays an important role in the pathobiology of these tumours. In a subset of 36 cases, coding regions of the p53 gene were completely sequenced via SSCP and direct DNA sequencing, revealing that overexpression of p53 protein is not always associated with point mutations in conserved exons of the p53 gene. Finally, we confirmed p53 protein expression in early-passage human glioma cell lines of known p53 mutational status and immunostaining scores. Although grade continues to be the strongest prognostic variable, the use of p53 staining as a prognostic indicator, in contrast to mutational DNA analyses, may be a useful adjunct in identifying patients at higher risk of treatment failure.

Alterations of the p53 tumour suppressor gene may be the most frequent abnormalities identified in human cancer. Early studies suggested that p53 abnormalities contribute to the development of malignant transformation in astrocytic brain tumours (Frankel *et al*, 1992; Sidransky *et al*, 1992). Loss of heterozygosity on human chromosome 17p, 53 point mutations, and altered p53 expression were reported in astrocytomas (Scheffner *et al*, 1990; Werness *et al*, 1990; Fujimaki *et al*, 1991; Lowe and Ruley, 1993; Chozick *et al*, 1994a; Iuzzolino *et al*, 1994; Slebos *et al*, 1994; Morita *et al*, 1996; Sarkar *et al*, 2002). Our goal is to assess the significance of potential alterations in p53 protein expression, and its inter-relationship with more traditional prognostic factors in astrocytoma tumour progression, in rigorous fashion.

Studies evaluating the prognostic significance of p53 abnormalities in human tumours were hampered initially by technical limitations imposed on detailed DNA mutational analyses using archival pathologic specimens. Monoclonal and polyclonal antibodies for p53 that reliably detect p53 protein in routine formalin-fixed histologic material have been developed. This approach revealed abnormalities, not only of mutant p53 protein, but also of apparently wild-type p53 protein (Barnes *et al*, 1992). Although most previous studies emphasised the importance of immunostaining as a screening procedure to identify patients with mutant p53 DNA alleles, immunostaining can also reveal an expanded spectrum of disease due to overexpression of nonmutant or wild-type p53 (Kurtkaya-Yapicier *et al*, 2002). In this report, we demonstrate the importance of overall p53 overexpression as an independent determinant of patient clinical core in astrocytic tumours. An expanded role for the importance of quantitative abnormalities in overall p53 expression is suggested by our finding that overexpression of p53 protein in astrocytic tumours may not be accompanied by detectable point mutations in coding exons of the p53 gene. The correlation we report between clinical progression-free survival and overexpression of p53, and its relationship to other clinical factors of importance in astrocytoma tumour progression, suggests that quantitative increases in expression of overall p53 protein are biologically significant in the neoplastic process of astrocytic tumours. Despite these intriguing statistical correlations, it should be noted that grade *per se* was the strongest prognostic factor, and remains the ‘gold standard’ in determining survival from astrocytic tumours.

## METHODS

### Patients

We examined a population of 74 patients with astrocytic tumours among patients treated for brain tumours at the Massachusetts General Hospital (MGH) between 1970 and 1990, for whom archival tissue were available and clinical outcome could be determined from medical records. The population characteristics of the cases were similar to those of the overall patient population evaluated at the MGH during the two decade period, as determined from MGH Tumor Registry data. In particular, the populations selected for study were indistinguishable with respect to overall tumour grade, patient age, gender, and the use of postoperative chemotherapy or radiation therapy. Actuarial overall survival was determined from the medical records as the time from pathologic confirmation of the surgical specimen to the time of death of the patient, with censoring occurring for intercurrent death from nontumour-related causes, and patients lost to follow-up. Progression-free survival was similarly determined as the time from pathologic confirmation of the surgical specimen, prior to therapy, to the first time of disease progression, as evidenced by a persistent 20-point decrease in Karnofsky functional status and available clinical and/or radiographic data. Survival end points were compared to standard prognostic factors that included patient age, sex, tumour grade, tumour size, Karnofsky score, and therapeutic interventions (extent of primary surgical debulking, radiation treatment, chemotherapy, and reoperation). All specimens examined were from the initial surgical specimen, prior to the administration of either radiation or chemotherapy. The tumour block deemed most representative of the overall surgical specimen was used for subsequent immunohistochemical analyses. In a subset of cases, additional blocks containing tumour tissue were also employed in immunohistochemical analyses in order to address potential heterogeneity in protein expression among the different paraffin blocks. Tumour grade was scored by the neuropathology staff at MGH prior to this study and those grades were confirmed by one neuropathologist at MGH MGH who rescored the tumour grades without knowledge of the p53 staining scores for the tissues (E Tessa Hedley-Whyte). Pathologic scoring was classified performed according to the World Health Organization Grading System (Radner *et al*, 2002). There were no pilocytic tumours in our clinicopathologic series. Therefore, stratification by pathologic criteria was grouped by ‘astrocytoma’ (WHO, grade II) *vs* anaplastic (malignant) astrocytoma and glioblastoma (WHO grades III,IV). Daumas-Duport criteria, as grades 1–4. Since the Daumas-Duport scale is only defined for adult supratentorial astrocytoma, 13 cases of astrocytoma, otherwise felt to be low grade by traditional histopathologic criteria, were also included in this study (Daumas-Duport *et al*, 1988). Mixed oligoastrocytic tumours were not included in this study because these types are not of pure astrocytic lineage.

### Immunohistochemistry

Immunohistochemical studies were performed on archival paraffin-embedded material from all 74 cases. In all, 10–15 5 *μ*m sections were obtained from each block. Normal horse serum (DAKO) was used to block slides for approximately 20 min prior to the application of antibody. Antibody PAb1801 (Crawford; UK; Oncogene Science, Uniondale, New York, USA) was used at dilutions of 1 : 125. Antibody incubations were conducted for 1 h at room temperature or overnight at 4°C. A biotinylated secondary antibody (Vector) recognising the primary antibody was applied for 20 min prior to the application of a streptavidin–peroxidase complex (Vector) for 30 min. Diaminobenzidine (Sigma) in phosphate-buffered saline was used as substrate for development of the peroxidase reaction. Owing to the preferences of the pathologists characterising the staining, slides were counterstained with haematoxylin. The brown immunoperoxidase reaction product was then easily discernable from the counterstain under regular illumination. In order to standardise immunostaining runs, each independent observer categorised the depth of staining as dark, medium, or light, and the quality of the staining as granular or diffuse. All scoring of cells was conducted at × 40 magnification. Oil immersion was rarely required for differentiation of immunoreactivity from nonspecific or counterstaining effects. Using these procedures, negative control slides containing paraffin-embedded Saos-2 cells (deleted in both p53 alleles) showed no staining over background, as verified by three independent pathologists. In addition, 20 individual autopsy cases containing normal cortex, midbrain, and brainstem showed no detectable immunostaining over background.

To ensure reproducibility, each case was stained at least twice. In a subset of cases, at least two blocks obtained from different regions of the respective tumours were evaluated in order to address issues of heterogeneity in protein expression. An additional round of staining occurred in cases with immunostaining scores differing by 1 score category (⩽25% of cells). Scoring of the immunostaining was performed independently by three observers. All of the cases were examined by all three observers in order to derive concordance scores for standardisation of the scoring categories. We included immunostaining categories as follows: (0) no detectable protein over background; (1) ⩽10% of cells staining; (2) >10–⩽25% of the cells staining; (3) >25–⩽50% of cells staining; (4) >50–⩽75% of cells staining; and (5) >75% of cells staining. In an attempt to address issues regarding nonspecific staining effects and batch-to-batch variability, note was taken of degrees of immunoreactivity by categorisation of the depth of the staining as dark, medium, or light, and the quality of the staining as granular or diffuse.

In order to assess p53 immunoreactivity using an antibody recognising a different epitope of p53, a second antibody was employed, D0-1 (Oncogene Science, Manhasset, NY, USA) 31 cases. As opposed to PAb 1801, an IgG1 monoclonal recognising residues 46–55 of human p53, antibody DO-1 is an IgG2a monoclonal recognising residues 21–25 of human p53. Following a preliminary series of experiments on control material, the optimal dilution for the batch of antibody used was determined as 1 : 50. The remainder of the immunohistochemistry procedure was as described above. Based on the spectrum of percent immunopositive cells, staining categories were determined as follows: (0) No immunoreactivity; (1) <1% of cells immunoreactive; (2)>1–⩽10% immunoreactive; (3) >10–⩽30% immunoreactive, (4) >30% immunoreactive.

### Absorption of p53 antibodies

All cases receiving staining scores greater than 0 on the percentage scale were also stained with preabsorbed PAb1801 antibody. Human recombinant mutant p53 (ARG 273 HIS) was expressed in *Escherichia Coli* strain BL2 (DE3)LysS transfected with human mutant p53 cDNA cloned into the T7 expression plasmid pT7-7 (from C Midgley and D Lane, University of Dundee, UK). Recombinant p53 was purified by immunoaffinity chromatography using antibody PAb240 and then coupled to CNBr-activated Sepharose 4B. A solution containing 100 mg of PAb1801 IgG and 0.2% gelatin was applied to the p53 protein-affinity column. The antibody solution was allowed to enter the column bed and flow was stopped. After 5 min, flow was resumed and fractions were collected. Fractions spanning the peak of total protein eluting from the column (representing the gelatin present in the starting material) were pooled.

The titre of the absorbed PAb1801 was determined by ELISA using 96-well microtitre plates coated with purified human recombinant p53. Absorbed and lot-matched unabsorbed antibodies were diluted using six serial 10-fold dilutions from 1 : 10^2^ to 1 : 10^7^ in dilution buffer (PBS containing 0.1% BSA). A volume of 100 *μ*l of each diluted antibody preparation was added to duplicate ELISA wells and incubated for 4 h at 37°C. Wells were washed and further incubated for 2 h at 37°C using 100 ml of peroxidase-conjugated goat anti-mouse IgG (Oncogene Science, Inc.) diluted to 2 mg ml^−1^ in dilution buffer. After washing, 100 *μ*l of peroxidase substrate was added to each well and the absorbance was determined at 405 nm (Bio-Tek Instruments Microplate reader, Model EL320). Antibody titre was defined as the reciprocal of dilutions rendering absorbances at 405 nm, 4 times background (background=0.024 AU). The residual PAb1801 activity, compared with the unabsorbed antibody, was found to be 0.05% for the same antibody lot used for immunohistochemistry.

### Evaluation of cytokeratin cross-reactivity

Cross-reactivity with keratin was reported in previous p53 immunostaining studies of astrocytomas (Sembritzki *et al*, 2002). Since cytokeratin immunoreactivity is recognised in gliomas and such immunoreactivity could confound results, all cases receiving p53 staining scores greater than 1 were also stained with a mixture of anticytokeratin antibodies consisting of AE1 and Cam 5.1 (Vector Laboratories). For positive tissue controls, a paraffin-embedded squamous cell carcinoma and a pituitary adenoma were compared. Negative control tissue included a malignant fibrous histiocytoma and a desmoid tumour. Approximately 10 *μ*l of each of the primary antibodies were employed at dilutions of 1 : 1000. The remainder of the immunohistochemistry procedure was performed as described above.

### p53 mutation analysis

In a subset of 36 tumours included in the study, DNA and RNA was harvested at the time of initial surgery. DNA extracted from tumour tissue, using standard procedures (phenol/chloroform and ethanol precipitation) was dissolved in 10 mM Tris, pH 7.5 and 1 mM EDTA and stored at 4°C. To screen for the presence of point mutations with the single-strand conformational polymorphism technique (SSCP), nine sets of primers were generated to amplify DNA fragments encompassing exon 2, and exons 4–11 of the p53 gene. The primer sequences and fragment lengths are described by Mashuyama (Mashuyama *et al*, 1991). The polymerase chain reaction (PCR) was performed using 100–500 ng of template DNA in 50 mM Tris-HCl, pH 8.6 with 1.5 mM MgCl_2_, 0.200 *μ*M of each dNTP, 250 ng of each primer, 1 ml of ^32^P-dCTP (3000 Ci mmol^−1^) diluted 1 : 10 and 2.5 U *Taq* polymerase (AmpliTaq, Cetus) in a 50 *μ*l total reaction volume. Reaction conditions for the Perkin-Elmer 480 Thermocycler were: 94°C (45 s), 55°C (45 s), and 72°C (45 s) for 35 cycles. The reaction was initiated with a 6 min ‘hot start’ incubation at 94°C, and was completed with a 7 min extension at 72°C, followed by 3 min at 92°C. A volume of 5 *μ*l of the PCR product were added to 5 *μ*l loading buffer (95% formamide, 20 mM EDTA, 0.05% bromophenol blue, and 0.05% xylene cyanol). The samples were denatured for 5 min at 85°C and loaded immediately into a 6% polyacrylamide-TBE nondenaturing gel containing 1–10% glycerol. Electrophoresis and autoradiography followed standard procedures. DNA samples determined to be abnormal by multiple SSCP analyses were amplified with the SSCP primers encompassing the abnormal region. Fragments were directly subcloned into PCR vector using the TA cloning TM system kit (In Vitrogen), and sequenced by the Sanger dideoxynucleotide method using a Sequenase 2.0 kit (US Biochemical). For each sample in which a base-pair alteration was identified, duplicate PCR reactions were performed to rule out the possibility of PCR-generated artefact.

### Statistical analyses

The following prognostic factors were derived from patient medical records and entered as independent variables into clinical databases: age, sex, initial tumour size, extent of surgical resection, radiation therapy dose and technique, chemotherapy, tumour grade, percentage of cells demonstrating p53 immunoreactivity, and Karnofsky performance status initial, postoperative preradiation or chemotherapy, following radiation/chemotherapy, and at time of last follow-up. Times to last clinical follow-up and disease-specific death were measured from the date of pathologic confirmation of the surgical specimen. In cases with multiple resections, only the tissue from the initial surgical resection, prior to the administration of radiation therapy or chemotherapy, was used for immunohistochemistry. The Kaplan – Meier Product-Limit method was used to estimate survival probabilities with statistical inferences on actuarial curves made using the log-rank test (Kaplan and Meier, 1958). Cox's proportional hazards regression was used to control for the simultaneous effect of outcome-related covariates (Cox, 1993). Martingale residual plots were used to assess model fit (Therneau *et al*, 1990). For all of our models, the ratio of hazards remained constant and the median follow-up time among censored patients was comparable between groups. Logistic regression and Fisher's exact two-tail test were used to examine the relationship between: p53 (low:<25% of the cells demonstrating immunoreactivity *vs* high:>25% of the cells positive) and the prognostic factors grade (1–2 *vs* 3–4), Karnofsky score (>70 *vs* ⩽70), age (⩽55 *vs* >55), size (⩽4 *vs* >4 cm), sex (female *vs* male), radiation dose (⩾6000 *vs* <6000 cGy), and chemotherapy (yes *vs* no). Since cutoff values were determined retrospectively, *P*-values were adjusted using the Bonferroni method (Bowker and Lieberman, 1972). Sensitivity and specificity were calculated with respect to a classification cutoff of *P*=0.50.

For survival analyses involving the subset of patients stained using Antibody DO-1, statistical analyses were conducted in identical manner. Since the greatest amount of immunoreactivity observed using this antibody approximated 50% of the cells staining positive, the p53 immunostaining categories differed as follows: p53 (low: <10% of the cells with immunoreactivity *vs* high: >10% of the cells staining positive).

## RESULTS

### p53 staining and patient outcome

**Figure 1:**
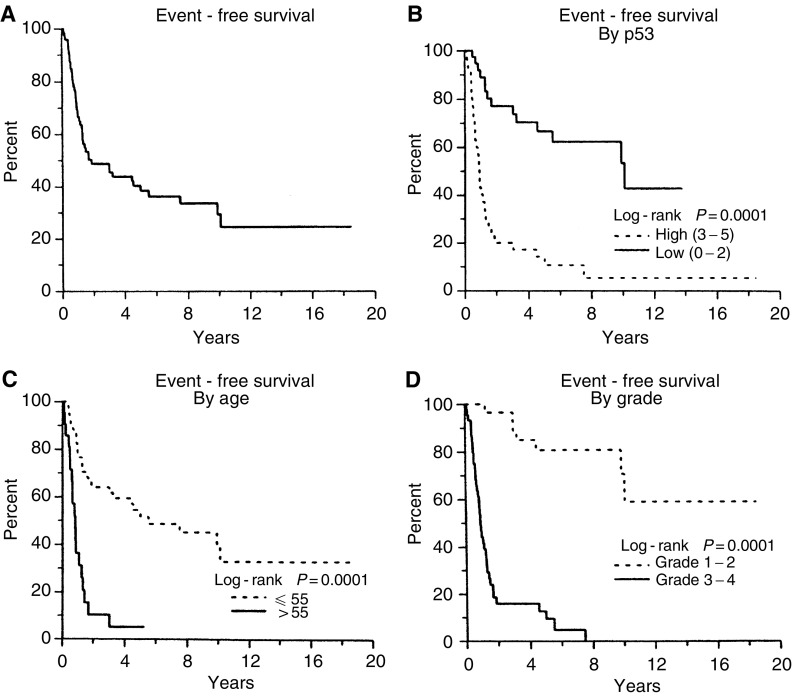
(**A**) Progression-free survival of the entire group of 74 patients. (**B**) Progression-free survival by grade. Grade I and II *vs* Grade III and IV. (**C**) Progression-free survival by age greater than or less than 55 years. (**D**) Progression-free survival by p53 expression. p53 high (at least 25–50% of the cells staining positive) *vs* P53 low (less than 25% of the cells staining positive).

Expression of p53 protein was compared to clinical outcome on both univariate and multivariate statistical analyses in order to determine whether the accumulation of p53 protein in tumour cells was important in the biological behaviour of astrocytomas. The clinical characteristics of the patient population are depicted in Figure 1

. Overall progression-free survival for the population of 74 patients is noted in Figure 1A. Interestingly, a low immunostaining score was a significant predictor of good clinical outcome (Figure 1B, *P*<0.0001). Similarly, increased tumour grade and patient age greater than 55 years correlated with poor clinical outcome (Figure 1C, D; *P*<0.001). This suggests that expression of p53 is a significant event in the natural history of astrocytic tumors.

Tumour grade, p53 immunostaining score, and patient age were the most significant prognostic factors using univariate Kaplan – Meier survival statistics (Table 1

**Table 1:** Relationship between p53 immunostaining and other prognostic factors (*N*=74)

	**Factor**	**Number of patients p53 low**	**Wald p53 high**	**Sensitivity *χ*^2^**	**Odds ratio (95% CI; *P*)**	**Specificity (*P*=0.50)**	**Fisher's (*P*=0.50)**	**Exact (two-tail)**
Grade	1, 2	25	4	18.8	15.4 (4.5–53.0; <0.01)	65.8%	88.9%	*P*⩽0.01
	3, 4	13	32					
Karnofsky	⩾70	16	5	6.7	4.5 (1.4–14.1; 0.010)	42.1%	86.1%	*P*=0.009
	⩽70	22	31					
Age	⩽55	32	21	5.7	3.8 (1.3–11.4; 0.020)	84.2%	41.7%	*P*=0.020
	>55	6	15					
Size	⩽4	14	5	4.8	3.6 (1.1–11.4; 0.029)	36.8%	86.1%	*P*=0.033
	>4	24	31					
Sex	Female	17	12	1.0	1.6 (0.63–4.1; 0.317)	44.7%	66.7%	*P*=0.349
	Male	21	24					
Radiation	>6000	6	3	0.9	2.1 (0.48–9.0; 0.334)	15.8%	0.0%	*P*=0.481
	⩽6000	32	33					
Chemotherapy	Yes	27	23	0.4	1.4 (0.52–3.7; 0.511)	71.1%	36.1%	*P*=0.511
	No	11	13					
Resection	Biopsy gross residual	8	2	3.3	4.5 (0.89–23.03; 0.068)	21.1%	94.4%	*P*=0.087
	Microscopic residual, total resection	30	34					

Prognostic factors for the overall population as stratified by p53 expression low (score 0–2) *vs* high (score 3–5): sex, Karnofsky performance status score, grade, extent of surgical resection, and the use of radiation therapy or chemotherapy.

). Other factors including tumour size, extent of surgical resection, Karnofsky index, radiation dose, use of chemotherapy, or patient sex did not predict patient outcome as well as p53 staining. The correlation of p53 staining scores with each prognostic factor is given in Table 2

**Table 2:** Kaplan – Meier statistics for the individual prognostic factors

**Variable**	**Wald *χ*^2^**	**Log rank *P***	**Survival ratio**
Grade [(1,2) *vs* (3,4)]	31.2	0.0001	15.7
P53 (low *vs* high)	25.4	0.0001	5.2
Age (⩽55 *vs* >55 years)	22.6	0.0001	4.7
Size (⩽4 *vs* >4 cm)	8.9	0.0014	3.7
Karnofsky (<70 *vs* ⩾70)	7.4	0.0047	2.8
Resection (biopsy, gross residual *vs* microscopic residual, total resection)	4.0	0.0035	3.3
Chemotherapy (no *vs* yes)	0.1	0.8039	1.1
Sex (male *vs* female)	0.0	0.9757	1.0

Progression-free survival (Kaplan – Meier model) (*N*=74, # events=46, % censored=37.84).

. Significant correlations were found when p53 expression was compared to tumour grade, Karnofsky score, patient age or tumour size. We examined such correlations with survival in detail using multivariate analyses to determine whether p53 expression was independent of other factors that predict clinical outcome in astrocytic tumours. Multivariate analyses demonstrated that the staining score was an independent variable in predicting clinical outcome (Table 3

**Table 3:** Multivariate analyses for the individual prognostic factors listed. p53 expression status rendered additional prognostic information when age and grade were controlled for in multivariate analyses

**Variable**	**Wald *χ*^2^**	***P*-value**	**Survival ratio**
Age (<55 *vs* >55 years)	4.0	0.0456	2.0
Grade (1–2 *vs* 3–4)	16.3	0.0001	8.9
p53 (low *vs* high)	4.1	0.0426	1.9
p53 (low *vs* high)	14.4	0.0001	3.7
Age (⩽55 *vs* >55 years)	8.7	0.0032	2.7
Size (⩽4 *vs* >4 cm)	2.6	0.1066	2.1
p53 (low *vs* high)	15.3	0.0001	4.0
Age (⩽55 *vs* >55 years)	11.7	0.0006	3.1
Resection (biopsy, gross residual *vs* microscopic residual, total resection)	0.2	0.6480	1.3

Progression-free survival (proportional-hazards model) (*N*=74, # events=46, % censored=37.84).

). In a proportional hazards model, p53 scores contributed additional predictive value for patient outcome when comparisons included tumour grade, patient age, tumour size, or Karnofsky score. Thus, the p53 protein immunoreactivity score did not merely reflect any other prognostic factor.

### Immunostaining

**Figure 2:**
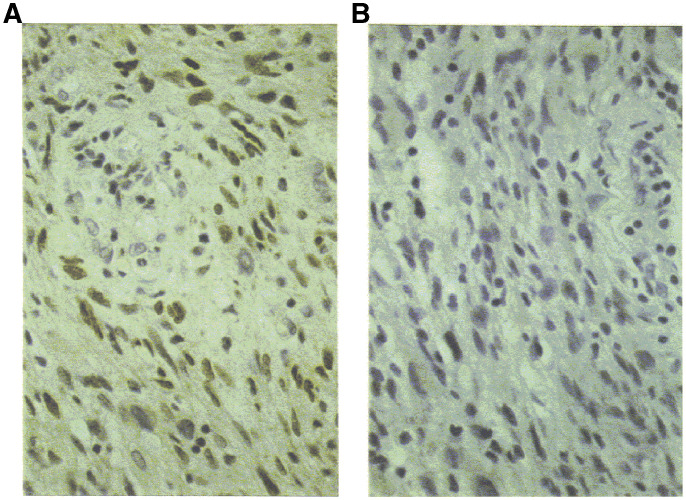
Shown is a grade 4/4 astrocytoma revealing a nuclear pattern of staining; however, following absorption of PAb1801, the nuclear staining decreases to background levels, thus demonstrating the specificity of the immunohistochemistry reaction.

The reliability of our immunohistochemical screening for p53, using the 1801 monoclonal antibody, was tested during this study. First, a cell line with both alleles of the p53 gene deleted (Saos-2) was included in all staining procedures to control for antibody dilutions. Second, none of the tumours with high immunostaining demonstrated cytokeratin immunoreactivity. Finally, we ensured that positively staining specimens were specifically expressing p53 by comparison to staining of the identical tumour specimens with an antibody that had been preabsorbed with recombinant p53; in all cases, the absorbed antibodies stained at background levels (Figure 2

). A striking finding in our study was the heterogeneity in protein expression found, not only among different tumours, but among different cells in histopathologic sections from the same specimen. In order to minimise heterogeneity in protein expression as a factor confounding the determination of the overall percentage of cells staining positive, each case was stained at least twice. In 62/70 cases, the staining score categories differed by no more than one score category. In five out of the eight remaining cases, a third or fourth staining run was required to obtain consistent pathologic findings. In the three remaining cases, the lowest of the staining scores was used for entry into clinical multivariate analyses. In addition, overall staining score concordance was reproducible in 66 out of 74 cases (89%). Thus, despite heterogeneity in expression, the staining score categories were quite reproducible among the three independent observers.

In order to assess the generality of our results with Ab 1801, another antibody, D0-1, was used in a subset of 31 cases judged to have sufficient remaining archival clinical material. The greatest amount of immunoreactivity observed using this antibody approximated 50% of the cells staining positive. Thus, the immunostaining categories reflect high p53 immunostaining as >10% of the cells staining positive. Using this criterion, overexpression of p53 was associated with decreased progression-free survival times on univariate analyses (*P*<0.04). Owing to the smaller sample size, however, statistical significance was not achieved on multivariate survival analyses.

### p53 staining and mutational analysis

**Figure 3:**
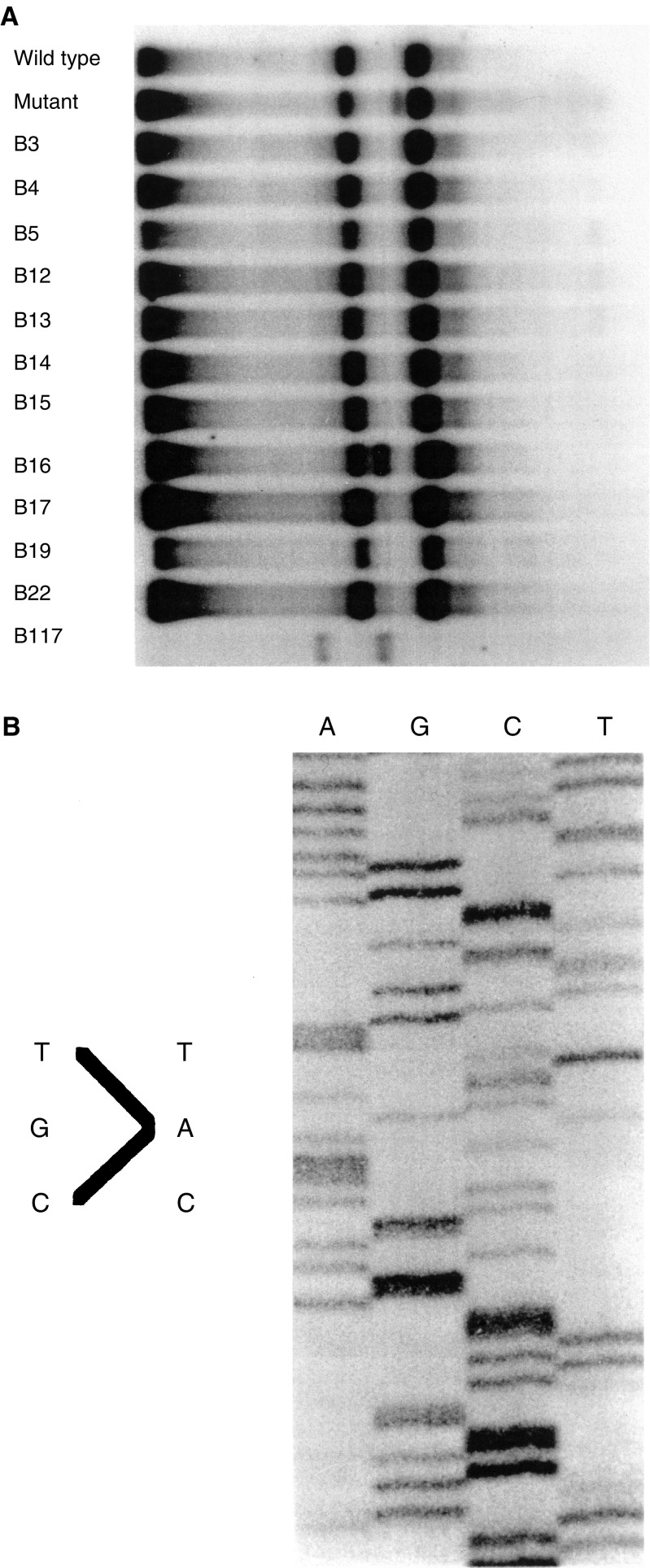
(**A**) A SSCP analysis of exon 8 of the p53 gene run on 12% polyacrylamide gel with 1% glycerol at 10 W for 16 h. Note band shift in lane marked B 16 (indicating a p53 alteration). The shift is a result of conformational changes induced by the point mutation identified in the sequencing gel in (**B**). (**B**) DNA sequence analysis of exon 8 for sample B 16 indicating base pair substitution at codon 273 changing an arginine residue to histidine. Nucleotide base pairs: A=adenine; G=guanine; C=cytosine; T=thymine.

Previous studies concentrated on the employment of immunostaining as a screening study to identify tumours containing p53 DNA mutations based on the premise that p53 mutations led to overexpression of p53 protein. In a subset of 36 patients we included in the immunostaining series, frozen tissue was available for molecular analyses. Additional molecular analyses were performed to assess the relationship between p53 mutation and p53 protein expression. The p53 coding sequences for exons 2–11 in DNA extracted from the primary tumours was completely sequenced in all of these cases. We found mutations in 13 out of these 36 tumours. All of these mutations were found in exons 4–8. Interestingly, one sample revealed a 10 bp deletion in exon 4, resulting in a stop codon. Although the sample corresponded to a patient with glioblastoma or Daumas-Duport grade 4/4 astrocytoma, the immunostaining data revealed <25% of cells positive, and an unusually long progression-free survival time of 20 months. An additional six cases overexpressing p53 protein showed no p53 DNA mutations. Thus, the data indicate that p53 overexpression can occur in the absence of mutations in coding sequences of the p53 gene, and suggests that overexpression of p53 protein, rather than p53 mutational status, is consistent with a statistically significant increase in progression-free survival times (Figure 3

).

Interestingly, the heterogeneity noted on protein expression studies was similarly noted on the DNA mutational level.

## DISCUSSION

It is well-recognised that alterations in p53 protein expression cause abnormalities in cell cycle regulation (Agami and Bernards, 2000). Normal p53 protein can block cells in the late G1 phase of the cycle, just prior to DNA synthesis, while mutant forms of this protein fail to arrest cell cycle progression and can promote cellular proliferation. Many studies of p53 expression stress the importance of the prolonged half-life of mutant p53 that allows protein accumulation in neoplastic tissue (Gannon *et al*, 1990). Several studies documented the importance of either wild-type or mutant p53 alleles in leading to increased expression of p53 protein increased expression of both wild-type and mutant p53 protein. Furthermore, the overexpression of wild-type p53 protein in human tumour specimens, as well as histologically benign lesions without p53 mutations, suggests a role for overexpression of p53 protein through overlapping mechanisms, idependent of actual p53 mutations (Barnes *et al*, 1992; Chozick *et al*, 1994b; Kurtkaya-Yapicier *et al*, 2002).

Our main goal was to determine, in rigorous fashion, whether abnormalities in p53 expression might play a role in the biologic outcome of patients with astrocytic brain tumours. We found that positive p53 staining was a significant predictor of poor clinical outcome in astrocytic tumours. As a predictor of outcome, p53 staining contributed to prognosis as an independent variable when compared with age, sex, tumour grade, Karnofsky score, or therapeutic approach. Although grade was the most statistically significant variable examined with respect to survival in astrocytomas, p53 expression was an independent predictor of patient survival. It should be noted that we were conservative in our statistical analyses of grade as a binary grouping (grades I–II *vs* grades III–IV). Indeed, in analyses where we examined grade as a continuous variable (I–IV), a higher level of statistical significance was achieved (data not shown). In like fashion, if we examine p53 protein expression as a continuous variable (all individual immunostaining score categories) rather than as a binary variable (⩽25 *vs* >25%), higher levels of statistical significance are once again achieved (data not shown). Even in this conservative setting, however, the levels of statistical significance achieved, independent of both grade and age, suggest that overexpression of p53 protein is important in the natural history of these aggressive tumours.

Finally, p53 expression was measured with an antibody recognising a different epitope of p53. Again, overexpression of p53 protein was associated with decreased progression-free survival times on univariate analyses. There was high concordance among cases overexpressing p53 with both antibodies. In all 9 out of 10 cases overexpressing p53 using Ab 1801, also overexpressed with Ab DO-1. Similarly, 14 of 16 cases demonstrating relatively low immunoreactivity using Ab DO-1 also showed low immunostaining scores using Ab 1801. Thus, 23 out of 26 cases revealed concordant immunostaining scores using either antibody. The fact that immunostaining categories with DO-1 differed from those established for Ab 1801, is not surprising given the lot and batch to batch variability of commercial preparations and the different antigen recognition site of the antibody. The technical complexity of archival studies emphasising various methodologic approaches to the interpretation of immunoreactivity was previously addressed (Fisher *et al*, 1994). Clearly, the absolute magnitude of standards for immunoreactivity will vary among investigators and clinical laboratories, though the relative degrees of immunostaining should be comparable.

We emphasised progression-free survival in our analyses, since we were primarily interested in assessing the interval of time during which the patient was free from significant neurologic deterioration due to tumour-related causes. This is particularly relevant with these rather heterogeneous tumour types, consisting of low-grade astrocytomas of rather long natural histories, and higher grade tumours with relatively short clinical progression-free intervals. While the differentiation of recurrent tumour *vs* radiation-induced morbidity can be complex, especially in the era preceding magnetic resonance imaging or positron emission tomography, a significant proportion of our patients underwent reoperation and/or necropsy within 1 month of their last clinical trial. Our identification of astrocytoma cases with high immunostaining scores, in the absence of mutations in coding sequences of the p53 gene, suggests that p53 may act through downstream mechanisms that are distinct from purely tumour-suppressive functions in its native form, or as a dominant-negative regulator in mutant or oncogenic forms. Normal cellular proteins that bind to p53 in a manner similar to viral oncoproteins were identified and found to be potential oncogenes (Scheffner *et al*, 1990; Bargonetti *et al*, 1991; Debbas and White, 1993; Nees *et al*, 2001; Cobbs *et al*, 2003; Dai *et al*, 2003; Calogero *et al*, 2004). Proteins implicated in cell cycle regulation may serve as downstream effectors of p53 function (Dulic *et al*, 1994; Agarwal *et al*, 1995; de Toledo *et al*, 1998; Skomedal *et al*, 1999; Ghimenti *et al*, 2003). The 1801 monoclonal antibody employed in our study detects a mammalian-specific epitope of both mutant and wild-type p53 protein. Although, based on the longer half-life of mutant p53, one might initially infer that most of the accumulation is due to overexpression of mutations in p53, overexpression of wild-type p53 would also be detected by this antibody (Gannon *et al*, 1990). Since the 1801 monoclonal antibody recognises total p53 expression, as was previously reported on archival paraffin-embedded preparations, we specifically chose to employ it in our studies. Although the so-called mutant-specific p53 antibodies exist, there is no antibody, to our knowledge, that recognises the entire mutational spectrum of p53 protein conformational changes. The discrimination of mutant *vs* wild-type p53 protein with the antibodies reported to date remains controversial (Fisher *et al*, 1994). More importantly, although prior reports emphasised the role of p53 mutation in leading to overexpression of p53 protein, the role of wild-type p53 overexpression in glial tumour progression has received considerably less attention (Rubio *et al*, 1993; Pollack *et al*, 2001; 2002). Several reports suggest that normal p53 protein may accumulate in tumours without DNA mutations, although, to our knowledge, no relationship among factors known to be of prognostic importance in astrocytic tumours has yet been described to establish clinical significance across the histologic spectrum of pure astrocytic tumours. Indeed, it is not surprising that p53 protein accumulates in non-neoplastic tissue or in the phenotypically normal tissue of cells from a cancer-prone family (Barnes *et al*, 1992; Kurtkaya-Yapicier *et al*, 2002; Mawrin *et al*, 2003).

A number of studies, besides our own, indicate inverse correlations among increased p53 expression and conventional factors indicating a favourable clinical course in astrocytomas (Chozick *et al*, 1994a; Iuzzolino *et al*, 1994; Korkolopoulou *et al*, 1998). Increased p53 protein was detected in a subset of low-grade *vs* high-grade astrocytomas (Chozick *et al*, 1994a). Increased p53 expression occurred in the recurrent *vs* the initial tumour pairs from the same patient, while tumours recurring within the same histologic grade category, failed to reveal this increase (Chozick *et al*, 1994a; Iuzzolino *et al*, 1994; Korkolopoulou *et al*, 1998; Sarkar *et al*, 2002). We excluded tumours of oligodendroglial or oligoastrocytic lineage from our analyses, because of the altered natural history as compared to ‘pure’ astrocytomas, thus increasing the likelihood of genetic mechanisms distinct from those of pure astrocytomas (Burger *et al*, 1987; Shaw *et al*, 1992). Interestingly, with respect to tumours of mixed or pure oligodendrocytic lineage, alterations in p53 expression may also portend a less favourable prognosis in the clinical setting (Soini *et al*, 1994; Korkolopoulou *et al*, 1997; Miettinen *et al*, 2001). Finally, pediatric tumours overexpressing p53 protein, revealed a worse prognosis with increasing age (Pollack *et al*, 2002).

Our data indicate an interesting relationship between age and p53 immunoreactivity in astrocytomas. In fact, while older age correlated with an increase in p53 immunoreactivity on univariate analyses, on multivariate analyses this effect conferred a rather modest overall statistical benefit in progression-free survival. Indeed, multivariate analyses indicated that the combination of low p53 immunostaining scores and young age rendered an approximate two-fold survival advantage. While this may seem impressive with respect to the natural history of low-grade tumours, the survival for high-grade astrocytomas and glioblastoma multiforme is frequently in the range of months, rather than years. The fact that multivariate analyses indicated an approximate nine-fold advantage with respect to grade *per se* helps place these statistical arguments into a clinical frame of reference (Table 3). Increased p53 immunoreactivity resulted in decreased survival with advancing age in a number of prior studies (Iuzzolino *et al*, 1994; Soini *et al*, 1994; Pollack *et al*, 2002). Of note, perhaps the larger of these indicated, much like the trend in our own data, that increased p53 immunoreactivity correlated with a worse prognosis in older children (Pollack *et al*, 2002). However, this study, with much larger numbers of young patient samples, as compared to our own, found that the interesting correlation between increased p53 expression and a worsened clinical course was age dependent. Older age correlated with increases in p53 expression and this correlation held up in multivariate analyses, even after controlling for the strongest traditional prognosticator in the clinical setting, histologic grade. The extent to which the inclusion of children less than 18 years of age is unknown. A total of 13 cases less than 18 years old were included in the study. Only two cases less than 3 years of age were included. Interestingly, both cases expressed high levels of p53 protein.

The existence of a subset of astrocytic tumours overexpressing p53 protein, in the absence of detectable p53 mutations, invokes a distinct role of apparently wild-type p53 expression in the natural history of astrocytomas (Burton *et al*, 2002). This result is not surprising given the spectrum of p53 function. An early study emphasised the importance of normal human p53 overexpression in the tumorigenic conversion of established fibroblasts (Tuck and Crawford, 1989). Similar classic studies described p53 as an antigen increased in SV40 virus-infected cells without p53 mutations (Bargonetti *et al*, 1991). Indeed, p53 expression is also increased in cells treated with DNA-damaging agents (Okada *et al*, 2003). Normal cellular proteins that bind to p53 in a manner similar to viral oncoproteins have been identified and found to be potential oncogenes (Nees *et al*, 2001). Alternatively, increased p53 protein expression might be representative of progressive chromosomal damage, such as cells treated *in vitro* with ultraviolet or gamma irradiation (Zhang *et al*, 1993; Agami and Bernards, 2000; Denko *et al*, 2000).

Both the genetic and environmental factors modulate p53 function. Genetic links are suggested via the decreased expression of p53 protein in a set of familial astrocytic tumours, as compared to sporadic cases from the same institution (Malmer *et al*, 2002). Three of our cases could be considered familial, as defined by the presence of nonpilocytic astrocytomas in first-degree relatives. In those six samples, revealing glioblastomas in both the patient and the first-degree relative, the p53 immunostaining scores indicated that between 25 and 50% of the cells stained positive. Genetics *per se* cannot distinguish familial aetiologies without considering other environmental factors including ethnicity and exposure to dietary or environmental changes (Chen *et al*, 2001). Despite a strong statistical correlation between our immunostaining results and prognostic factors of importance in astrocytoma tumour progression, further study is necessary before p53 immunostaining is routinely used in a prospective clinical setting. First of all, selection bias cannot be completely eliminated in any retrospective study, even with statistical analyses substantiating similarities to the clinical population evaluated at the same institution during the same time period. The use of the Bonferroni statistical correction only partially compensates for the retrospective nature of the analyses (Bowker and Lieberman, 1972). Although p53 expression yielded additional information with respect to prognosis when the two statistically strongest prognostic factors (i.e. grade and age) were controlled for in multivariate analyses, we must emphasise that grade *per se* remains the most important prognostic factor with respect to actuarial survival on both univariate and multivariate analyses. Multivariate analyses indicate low p53 immunostaining scores and young age to render an approximate two-fold survival advantage; however, low-grade histology *per se* yielded an approximate nine-fold advantage (Table 3). Thus, the role of p53 expression in patient evaluation should be evaluated in conjunction with more traditional indices of glial tumour prognosis, including patient age, and grade.

In summary, overexpression of total p53 protein, both mutant and wild type, plays an important role in the natural history of patients with astrocytic tumours. This observation suggests that immunostaining of astrocytomas for overall p53 protein production at diagnosis may be a valuable tool in the determination of clinical course and be helpful in better tailoring modalities of therapeutic intervention to the individual clinical case at hand. Overexpression of p53 protein may not be associated with mutations in coding exons of the p53 gene. Further studies are needed in order to formulate a mechanistic explanation for the enhanced accumulation of p53 protein in astrocytic tumours. Elucidation of these mechanisms should further our understanding of the clinical biology of astrocytic neoplasms, and hopefully assist in improving therapeutic approaches for these aggressive tumours.

## References

[bib1] Agami R, Bernards R (2000) Distinct initiation and maintenance mechanisms cooperate to induce G1 cell cycle arrest in response to DNA damage. Cell 102: 55–66 10929713 10.1016/s0092-8674(00)00010-6

[bib59] Agarwal ML, Agarwal A, Taylor WR, Stark GR (1995) p53 controls both the G2/M and the G1 cell cycle checkpoints and mediates reversible growth arrest in human fibroblasts. Proc Natl Acad Sci USA 92: 8493–8497 7667317 10.1073/pnas.92.18.8493 PMC41183

[bib2] Bargonetti J, Friedman PN, Kern SE, Vogelstein B, Prives C (1991) Wild-type but not mutant p53 immunopurified proteins bind to sequences adjacent to the SV40 origin of replication. Cell 65: 1083–1091 1646078 10.1016/0092-8674(91)90560-l

[bib3] Barnes DM, Hanby AM, Gillett CE, Mohammed S, Hodgson S, Bobrow LG, Leigh IM, Purkis T, MacGeoch C, Spurr NK, Bartek J, Vojtesek B, Picksley SM, Lane DP (1992) Abnormal expression of wild type p53 protein in normal cells of a cancer family patient. Lancet 340: 259–263 1353190 10.1016/0140-6736(92)92354-i

[bib4] Bowker A, Lieberman G (1972) Engineering Statistics. Prentice-Hall: NJ

[bib5] Burger PC, Rawlings CE, Cox EB, McLendon RE, Schold Jr SC, Bullard DE (1987) Clinicopathologic correlations in the oligodendroglioma. Cancer 59: 1345–1352 3545437 10.1002/1097-0142(19870401)59:7<1345::aid-cncr2820590719>3.0.co;2-a

[bib6] Burton EC, Lamborn KR, Forsyth P, Scott J, O'Campo J, Uyehara-Lock J, Prados M, Berger M, Passe S, Uhm J, O'Neill BP, Jenkins RB, Aldape KD (2002) Aberrant p53, mdm2, and proliferation differ in glioblastomas from long-term compared with typical survivors. Clin Cancer Res 8: 180–187 11801556

[bib42] Calogero A, Lombari V, De Gregorio G, Porcellini A, Ucci S, Arcella A, Caruso R, Gagliardi FM, Gulino A, Lanzetta G, Frati L, Mercola D, Ragona G (2004) Inhibition of cell growth by EGR-1 in human primary cultures from malignant glioma. Cancer Cell Int 4: 1 14711380 10.1186/1475-2867-4-1 PMC324562

[bib43] Carozza SE, Wrensch M, Miike R, Newman B, Olshan AF, Savitz DA, Yost M, Lee M (2000) Occupation and adult gliomas. Am J Epidemiol 152: 838–846 11085395 10.1093/aje/152.9.838

[bib44] Chen PC, Aldape K, Wiencke JK, Kelsey KT, Miike R, Davis RL, Liu J, Kesler-Diaz A, Takahashi M, Wrensch M (2001) Ethnicity delineates different genetic pathways in malignant glioma. Cancer Res 61: 3949–3954 11358811

[bib7] Chozick BS, Pezullo JC, Epstein MH, Finch PW (1994a) Prognostic implication of p53 overexpression in supratentorial astrocytic tumors. Neurosurgery 35: 831–838 7838330 10.1227/00006123-199411000-00005

[bib8] Chozick BS, Weicker ME, Pezzullo JC, Jackson CL, Finkelstein SD, Ambler MW, Epstein MH, Finch PW (1994b) Pattern of mutant p53 expression in human astrocytomas suggests the existence of alternate pathways of tumorigenesis. Cancer 73: 406–415 8293408 10.1002/1097-0142(19940115)73:2<406::aid-cncr2820730228>3.0.co;2-s

[bib45] Cobbs CS, Whisenhunt TR, Wesemann DR, Harkins LE, Van Meir EG, Samanta M (2003) Inactivation of wild-type p53 protein function by reactive oxygen and nitrogen species in malignant glioma cells. Cancer Res 63: 8670–8673 14695179

[bib9] Cox DR (1993) Regression models and life-tables. J R Stat Soc Bull 34: 187–220

[bib46] Dai S, Huang ML, Hsu CY, Chao KS (2003) Inhibition of hypoxia inducible factor 1alpha causes oxygen-independent cytotoxicity and induces p53 independent apoptosis in glioblastoma cells. Int J Radiat Oncol Biol Phys 55: 1027–1036 12605983 10.1016/s0360-3016(02)04507-8

[bib47] Das A, Tan WL, Teo J, Smith DR (2002) Glioblastoma multiforme in an Asian population: evidence for a distinct genetic pathway. J Neurooncol 60: 117–125 12635658 10.1023/a:1020622415786

[bib10] Daumas-Duport C, Scheithauer B, O'Fallon J, Kelly P (1988) Grading of astrocytomas. A simple and reproducible method. Cancer 62: 2152–2165 3179928 10.1002/1097-0142(19881115)62:10<2152::aid-cncr2820621015>3.0.co;2-t

[bib48] de Toledo SM, Azzam EL, Keng P, Laffrenier S, Little JB (1998) Regulation by ionizing radiation of CDC2, cyclin A, cyclin B, thymidine kinase, topoisomerase IIalpha, and RAD51 expression in normal human diploid fibroblasts is dependent on p53/p2lWafl. Cell Growth Differ 9: 887–896 9831241

[bib40] Debbas M, White E (1993) Wild-type p53 mediates apoptosis by E1A, which is inhibited by E1B. Genes Dev 7: 546–554 8384580 10.1101/gad.7.4.546

[bib11] Denko NC, Green SL, Edwards D, Giaccia AJ (2000) p53 checkpoint-defective cells are sensitive to X rays, but not hypoxia. Exp Cell Res 258: 82–91 10912790 10.1006/excr.2000.4928

[bib49] Dulic V, Kaufmann WK, Wilson SJ, Tisty TD, Lees E, Harper JW, Elledge SJ, Reed SI (1994) p53-dependent inhibition of cyclin-dependent kinase activities in human fibroblasts during radiation-induced G1 arrest. Cell 76: 1013–1023 8137420 10.1016/0092-8674(94)90379-4

[bib12] Fisher CJ, Gillett CE, Vojtesek B, Barnes DM, Millis RR (1994) Problems with p53 immunohistochemical staining: the effect of fixation and variation in the methods of evaluation. Br J Cancer 69: 26–31 7506924 10.1038/bjc.1994.4 PMC1968757

[bib13] Frankel RH, Bayona W, Koslow M, Newcomb EW (1992) p53 mutations in human malignant gliomas: comparison of loss of heterozygosity with mutation frequency. Cancer Res 52: 1427–1433 1347252

[bib14] Fujimaki T, Matsutani M, Nakamura O, Asai A, Funada N, Koike M, Segawa H, Aritake K, Fukushima T, Houjo S, Tamura A, Sano K (1991) Correlation between bromodeoxyuridine-labeling indices and patient prognosis in cerebral astrocytic tumors of adults. Cancer 67: 1629–1634 2001552 10.1002/1097-0142(19910315)67:6<1629::aid-cncr2820670626>3.0.co;2-e

[bib15] Gannon JV, Greaves R, Iggo R, Lane DP (1990) Activating mutations in p53 produce a common conformational effect. A monoclonal antibody specific for the mutant form. EMBO J 9: 1595–1602 1691710 10.1002/j.1460-2075.1990.tb08279.x PMC551855

[bib50] Ghimenti C, Fiano V, Chiado-Piat L, Chio A, Cavalla P, Schiffer D (2003) Deregulation of the p14ARF/Mdm2/p53 pathway and G1/S transition in two glioblastoma sets 77. J Neurooncol 61: 95–102 12622447 10.1023/a:1022127302008

[bib16] Iuzzolino P, Ghimenton C, Nicolato A, Giorgiutti F, Fina P, Doglioni C, Barbareschi M (1994) p53 protein in low grade astrocytomas: a study with long-term follow-up. Br J Cancer 69: 586–591 8123492 10.1038/bjc.1994.107 PMC1968882

[bib17] Kaplan EL, Meier P (1958) Nonparametric estimation from incomplete observations. J Am Stat Assoc 53: 457–481

[bib18] Korkolopoulou P, Christodoulou P, Kouzelis K, Hadjiyannakis M, Priftis A, Stamoulis G, Seretis A, Thomas-Tsagli E (1997) MDM2 and p53 expression in gliomas: a multivariate survival analysis including proliferation markers and epidermal growth factor receptor. Br J Cancer 75: 1269–1278 9155045 10.1038/bjc.1997.216 PMC2228241

[bib19] Korkolopoulou P, Kouzelis K, Christodoulou P, Papanikolaou A, Thomas-Tsagli E (1998) Expression of retinoblastoma gene product and p21 (WAF1/Cip 1) protein in gliomas: correlations with proliferation markers, p53 expression and survival. Acta Neuropathol (Berl) 95: 617–624 9650754 10.1007/s004010050848

[bib51] Krishnan G, Felini M, Carozza SE, Miike R, Chew T, Wrensch M (2003) Occupation and adult gliomas in the San Francisco Bay Area. Journal of Occupational and Environmental Medicine 45: 637–647 10.1097/01.jom.0000069245.06498.48 12802217

[bib20] Kurtkaya-Yapicier O, Scheithauer BW, Hebrink D, James CD (2002) p53 in nonneoplastic central nervous system lesions: an immunohistochemical and genetic sequencing study. Neurosurgery 51: 1246–1254 12383370 10.1097/00006123-200211000-00021

[bib21] Lowe SW, Ruley HE (1993) Stabilization of the p53 tumor suppressor is induced by adenovirus 5 E1A and accompanies apoptosis. Genes Dev 7: 535–545 8384579 10.1101/gad.7.4.535

[bib52] Malmer B, Brannstrom T, Andersson U, Bergh K, Gronberg H, Henriksson R (2002) Does a low frequency of P53 and Pgp expression in familial glioma compared to sporadic controls indicate biological differences? Anticancer Res 22: 3949–3954 12553017

[bib22] Mashuyama S, Murikami Y, Yoshimoto T, Sekiya T, Hayashi K (1991) Detection of p53 gene mutation in human brain tumors by single strand confinement polymorphism: analysis of polymerase chain-reaction products. Oncogene 6: 1313–1318 1886708

[bib53] Mawrin C, Lins H, Kirches E, Schildhaus HU, Scherlach C, Kanakis D, Dietzmann K (2003) Distribution of p53 alterations in a case of gliomatosis cerebri 84. Hum Pathol 34: 102–106 12605375 10.1053/hupa.2003.1

[bib23] Miettinen HE, Paunu N, Rantala I, Kalimo H, Paljarvi L, Helin H, Haapasalo H (2001) Cell cycle regulators (p21, p53, pRb) in oligodendrocytic tumors: a study by novel tumor microarray technique. J Neurooncol 55: 29–37 11804280 10.1023/a:1012961918848

[bib24] Morita M, Rosenblum MK, Bilsky MH, Fraser RAR, Rosenfeld MR (1996) Long-term survivors of glioblastoma multiforme: clinical and molecular characteristics. J Neuro Oncol 27: 259–266 10.1007/BF00165483 8847560

[bib25] Nees M, Geoghegan JM, Hyman T, Frank S, Miller L, Woodworth CD (2001) Papillomavirus type 16 oncogenes downregulate expression of interferon-responsive genes and upregulate proliferation-associated and NF-kappaB-responsive genes in cervical keratinocytes. J Virol 75: 4283–4296 11287578 10.1128/JVI.75.9.4283-4296.2001 PMC114174

[bib26] Okada Y, Hurwitz EE, Esposito JM, Brower MA, Nutt CL, Louis DN (2003) Selection pressures of TP53 mutation and microenvironmental location influence epidermal growth factor receptor gene amplification in human glioblastomas. Cancer Res 63: 413–416 12543796

[bib54] Pardo FS, Aronen HG, Kennedy D, Skates S, Paiva K, Okunieff P, Schmidt EV, Hochberg FH, Harsh GR, Fischman AJ, Linggood RM, Rosen BR (1994) Functional cerebral imaging in the evaluation and radiotherapeutic treatment planning of patients with malignant glioma. Int J Rad Oncol Biol Phys 30: 663–669 10.1016/0360-3016(92)90953-f 7928498

[bib60] Pollack IF, Finkelstein SD, Burnham J, Holmes EJ, Hamilton RL, Yates AJ, Finlay JL, Sposto R (2001) Age and TP53 mutation frequency in childhood malignant gliomas: results in a multi-institutional cohort. Cancer Res 61: 7404–7407 11606370

[bib27] Pollack IF, Finkelstein SD, Woods J, Burnham J, Holmes EJ, Hamilton RL, Yates AJ, Boyett JM, Finlay JL, Sposto R (2002) Expression of p53 and prognosis in children with malignant gliomas. N Engl J Med 346: 420–427 11832530 10.1056/NEJMoa012224

[bib55] Rubio M-P, von Deimling A, Yandell DW, Wiestler OD, Guisella JF, Louis DN (1993) Accumulation of wild type p53 protein in human astrocytomas. Cancer Res 53: 3465–3467 8339248

[bib28] Radner H, Blumcke I, Reifenberger G, Wiestler OD (2002) [The new WHO classification of tumors of the nervous system 2000. Pathology and genetics]. Pathologe 23: 260–283 12185780 10.1007/s00292-002-0530-8

[bib29] Sarkar C, Ralte AM, Sharma MC, Mehta VS (2002) Recurrent astrocytic tumours – a study of p53 immunoreactivity and malignant progression. Br J Neurosurg 16: 335–342 12389885 10.1080/02688697021000007588

[bib30] Scheffner M, Werness BA, Huibregtse JM, Levine AJ, Howley PM (1990) The E6 oncoprotein encoded by human papillomavirus types 16 and 18 promotes the degradation of p53. Cell 63: 1129–1136 2175676 10.1016/0092-8674(90)90409-8

[bib31] Sembritzki O, Hagel C, Lamszus K, Deppert W, Bohn W (2002) Cytoplasmic localization of wild-type p53 in glioblastomas correlates with expression of vimentin and glial fibrillary acidic protein. Neurooncology 4: 171–178 10.1093/neuonc/4.3.171 PMC1920636 12084347

[bib32] Shaw EG, Scheithauer BW, O'Fallon JR, Tazelaar HD, Davis H (1992) Oligodendrogliomas: the Mayo Clinic experience. J Neurosurg 76: 428–434 1738022 10.3171/jns.1992.76.3.0428

[bib33] Sidransky D, Mikkelsen T, Schwechhekner K, Rosenblum M, Cavanee W, Vogelstein B (1992) Clonal expansion of p53 mutant cells is associated with brain tumour progression. Nature 355: 846–847 1311419 10.1038/355846a0

[bib56] Skomedal H, Kristensen GB, Lie AK, Holm R (1999) Aberrant expression of the cell cycle associated proteins TP 53, MDM2, p21, p27, cdk4, cyclin Dl, RB, and EGFR in cervical carcinomas. Gynecol Oncol 73: 223–228 10329038 10.1006/gyno.1999.5346

[bib34] Slebos RJC, Lee MH, Plunkett BS, Kessis TD, Williams BO, Jacks T, Hedrick L, Kastan MB, Cho KR (1994) p53-dependent G_1_ arrest involves pRB-related proteins and is disrupted by the human papillomavirus 16 E7 oncoprotein. Proc Natl Acad Sci USA 91: 5320–5324 8202487 10.1073/pnas.91.12.5320 PMC43986

[bib35] Soini Y, Niemela A, Kamel D, Herva R, Bloigu R, Paakko P, Vahakangas K (1994) p53 immunohistochemical positivity as a prognostic marker in intracranial tumours. APMIS 102: 786–792 7826609 10.1111/j.1699-0463.1994.tb05235.x

[bib57] Tedeschi-Blok N, Schwartzbaum J, Lee M, Miike R, Wrensch M (2001) Dietary calcium consumption and astrocytic glioma: The San Francisco Bay Area Adult Glioma Study, 1991–1995. Nutr Cancer – An Int J 39: 196–203 10.1207/S15327914nc392_6 11759280

[bib36] Therneau TM, Grambsch PM, Fleming TR (1990) Martingale-based residuals and survival models. Biometrika 77: 147–160

[bib37] Tuck SP, Crawford L (1989) Overexpression of normal human p53 in established fibroblasts leads to their tumorigenic conversion. Oncogene Res 4: 81–96 2654816

[bib38] Werness BA, Levine AJ, Howley PM (1990) Association of human papillomavirus types 16 and 18 E6 proteins with p53. Science 248: 76–79 2157286 10.1126/science.2157286

[bib58] Wiemels JL, Wiencke JK, Sison JD, Miike R, McMillan A, Wrensch M (2002) History of allergies among adults with glioma and controls. Int J Cancer 98: 609–615 11920623 10.1002/ijc.10239

[bib39] Zhang W, Shay JW, Deisseroth A (1993) Inactive p53 mutants may enhance the transcriptional activity of wild-type p53. Cancer Res 53: 4772–4775 8402659

